# Stereoselective Covalent Adduct Formation of Acyl Glucuronide Metabolite of Nonsteroidal Anti-Inflammatory Drugs with UDP-Glucuronosyltransferase

**DOI:** 10.3390/ijms23094724

**Published:** 2022-04-25

**Authors:** Atsushi Kawase, Rio Yamashita, Tsubasa Yoshizato, Mashiro Yoshikawa, Hiroaki Shimada, Masahiro Iwaki

**Affiliations:** 1 Department of Pharmacy, Faculty of Pharmacy, Kindai University, Osaka 577-8502, Japan; 1933420013y@kindai.ac.jp (R.Y.); 2133420012g@kindai.ac.jp (T.Y.); 1811710035y@kindai.ac.jp (M.Y.); shimada@phar.kindai.ac.jp (H.S.); kindai20@gmail.com (M.I.); 2 Pharmaceutical Research and Technology Institute, Kindai University, Osaka 577-8502, Japan; 3 Antiaging Center, Kindai University, Osaka 577-8502, Japan

**Keywords:** NSAIDs, glucuronidation, glucuronide, stereoselective, liver injury, covalent adduct, endoplasmic reticulum

## Abstract

A reactive metabolite of nonsteroidal anti-inflammatory drugs (NSAIDs), acyl-β-D-glucuronide (AG), covalently binds to endogenous proteins. The covalent adduct formation of NSAIDs-AG may lead to the dysfunction of target proteins. Therefore, it is important to clarify the detailed characterization of the formation of covalent protein adducts of NSAID-AG. UDP-glucuronosyltransferase (UGT) catalyzes the conversion of NSAIDs to NSAIDs-AG. The aim of this study was to perform a quantitative analysis of the covalent adduct formation of NSAIDs-AG with UGT. Diclofenac-AG and ketoprofen-AG formed covalent adducts with organelle proteins. Next, the number of covalent adducts formed between NSAIDs-AG and UGT isoforms (UGT1A1, UGT1A9, UGT2B4, and UGT2B9) was determined. The capacity of diclofenac-AG to form covalent adducts with UGT1A9 or UGT2B7 was approximately 10 times higher than that of mefenamic acid-AG. The amounts of covalent adducts of AG of propionic acid derivative NSAIDs with UGT2B were higher than those with UGT1A. Stereoselectivity was observed upon covalent binding to UGT. A significant negative correlation between the half-lives of NSAIDs-AG in phosphate buffers and the amount of covalent adduct with UGT2B7 was observed, suggesting the more labile NSAID-AG forms higher irreversible bindings to UGT. This report provides comprehensive information on the covalent adduct formation of NSAIDs-AGs with UGT.

## 1. Introduction

UDP-glucuronosyltransferase (UGT), which catalyzes glucuronidation reactions for hydroxy, carboxy, amino, and thiol groups of substrates, exists in the endoplasmic reticulum (ER). Nonsteroidal anti-inflammatory drugs (NSAIDs) containing carboxylic acids undergo glucuronidation reactions so as to be converted to NSAIDs-acyl-β-D-glucuronide (NSAIDs-AG). NSAIDs-AG, which is well known as an electrophilic reactive metabolite of NSAIDs, covalently binds endogenous proteins in livers and plasma [1,2,3,4]. NSAIDs-AG interacts with nucleophilic residues of proteins. NSAIDs-AG forms covalent adducts via two mechanisms: transacylation (direct nucleophilic displacement) and glycation mechanisms. In the glycation mechanism, isomers of NSAIDs-AG react with amino groups of the protein after acyl migration in NSAIDs-AG. For example, diclofenac (DIC)-AG covalently binds endogenous proteins in bile canaliculus depending on ABCC2 function [3]. Furthermore, NSAIDs-AG forms covalent protein adducts with canalicular membrane proteins, such as dipeptidyl peptidase IV after excretion into the bile via efflux transporters (such as ABCC2 and ABCG2) [5]. There is a possibility that the covalent adduct formation of NSAIDs-AG leads to dysfunction of target proteins and the acquisition of antigenicity [6]. Therefore, it is important to clarify the detailed characterization of the formation of covalent protein adducts of NSAIDs-AG.

**Figure 1:**
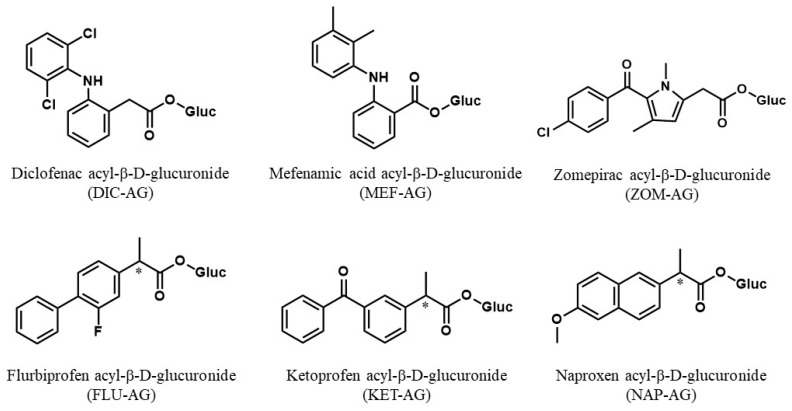
Chemical structures of NSAIDs-acyl-β-D-glucuronide (NSAIDs-AG). AG of diclofenac (DIC); phenylacetic acid derivative, mefenamic acid (MEF); fenamic acid derivative, zomepirac (ZOM); benzoylpyrrole acetic acid derivative, flurbiprofen (FLU), ketoprofen (KET), and naproxen (NAP); propionic acid derivatives were used. * indicates chiral center.

Previous studies demonstrated that ketoprofen (KET)-AG formed a covalent adduct with UGT [7]. However, the quantitative analysis of covalent adduct formation of NSAIDs-AG with UGT has not been determined. Stereoselective covalent adduct formation of NSAIDs-AG with human serum albumin (HSA) has been reported [8,9,10]. Therefore, we investigated whether other NSAIDs-AG and KET-AG form covalent adducts with UGT, whether there are differences in covalent adduct formation of NSAIDs-AG between UGT1A and UGT2B, and whether there is stereoselectivity in covalent adduct formations with UGT. We chose UGT isoforms UGT1A1, UGT1A9, UGT2B4, and UGT2B7 because UGT1A9 and UGT2B7 predominantly participate in the glucuronidation of NSAIDs in humans, whereas UGT1A1 and UGT2B4 are abundantly expressed in the liver. We chose NSAIDs DIC, mefenamic acid (MEF), zomepirac (ZOM), *R*-flurbiprofen (*R*-FLU), *S*-FLU, *R*-KET, *S*-KET, *R*-naproxen (*R*-NAP), and *S*-NAP. Four drug-induced liver injury (DILI)-associated types of NSAIDs, derivatives of aryl acetic acid, fenamic acid, pyrrole-acetic acid, and propionic acid, are also expressed. This study provides quantitative information on the covalent adduct formation of NSAIDs-AG used in this study (Figure 1) with endogenous proteins such as UGT.

## 2. Results

### 2.1. Concentration- and Time-Dependent Formation of Covalent Adducts

**Figure 2:**
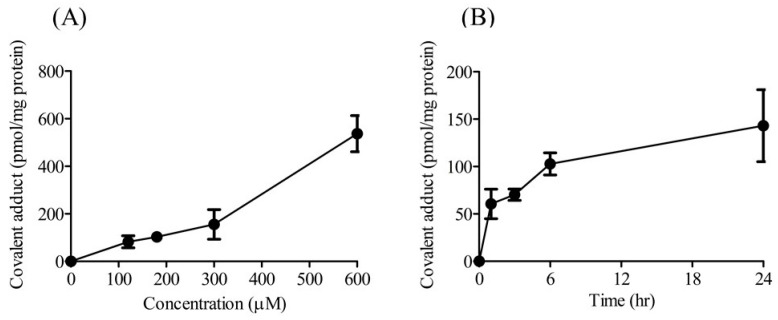
Covalent adduct formation of DIC-AG with the proteins of Mic in various concentrations of DIC-AG for 6 h (**A**) and time course at 180 μM of DIC-AG (**B**). (**A**) DIC-AG at the concentrations of 120, 180, 300, and 600 μM and (**B**) sampling times of 1, 3, 6, and 24 h. The results are expressed as the mean ± SD of each group (*n* = 3–5).

The effects of DIC-AG concentration and incubation time on the formation of covalent adducts of DIC-AG with Mic proteins were examined so as to optimize the experimental conditions. The number of covalent adducts of DIC-AG with the proteins of Mic increased in a concentration- and time-dependent manner (Figure 2). NSAIDs-AG concentrations and incubation times were set at 180 μM and 6 h, respectively.

### 2.2. Covalent Adduct Formation with Organelle Proteins

**Figure 3:**
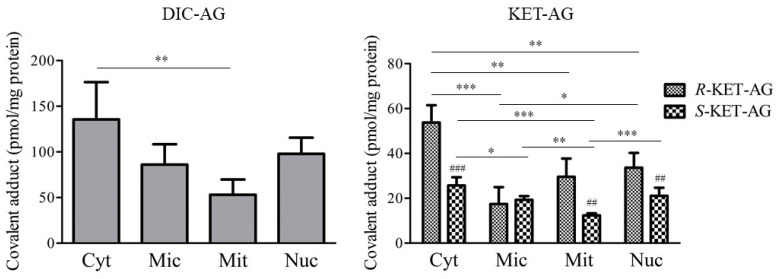
Covalent adduct formation of DIC-AG, *R*-KET-AG, and *S*-KET-AG with organelle proteins of Cyt, Mic, Mit, and Nuc. DIC-AG, *R*-KET-AG, or *S*-KET-AG were incubated with Cyt, Mic, Mit, and Nuc for 6 h. The results are expressed as the mean ± SD of each group (*n* = 3–5). Statistical analysis was performed using the Bonferroni test. *, **, and *** indicate significant differences among organelle proteins with *p* < 0.05, *p* < 0.01, and *p* < 0.001, respectively. ##, and ### indicate significant differences between *R* and *S* diastereomer with *p* < 0.01, and *p* < 0.001, respectively.

To characterize the covalent adduct formation of NSAIDs-AG with organelle proteins, the amounts of covalent adducts of DIC-AG, *R*-KET-AG, and *S*-KET-AG with Cyt, Mic, Mit, and Nuc were measured (Figure 3). Both DIC-AG and KET-AG formed covalent adducts with proteins and informed all fractions of the liver homogenate. Higher amounts of covalent adducts with Cyt proteins were observed than those with Mit proteins. When comparing the diastereomers of KET-AG and *R*-KET-AG, higher amounts of covalent adducts were formed with proteins Cyt, Mit, and Nuc. There was no formation of covalent adducts of NSAIDs-AG in the incubation conditions without organelle proteins (data not shown).

### 2.3. Covalent Adduct Formation with UGTs

**Figure 4:**
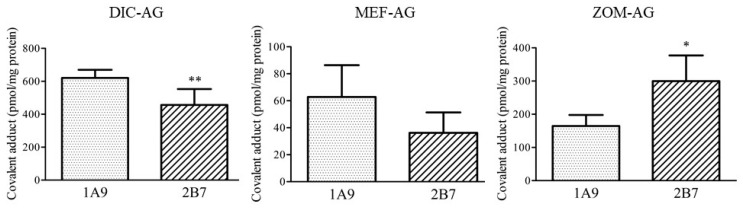
Covalent adduct formation of DIC-AG, MEF-AG, and ZOM-AG with UGT1A9 and UGT2B7. NSAIDs-AG were incubated with UGT1A9 and UGT2B7 for 6 h. The results are expressed as the mean ± SD of each group (*n* = 5). Statistical analysis was performed using Student’s *t*-test. Significant differences (* *p* < 0.05 and ** *p* < 0.01) between UGT1A9 and UGT2B7 were observed.

Next, the formation of covalent adducts of NSAIDs-AG with the UGTs was examined. The covalent adducts of AG in DIC as an aryl acetic acid derivative, MEF as a fenamic acid derivative, and ZOM as a pyrrole-acetic acid derivative were examined (Figure 4). Among the UGT isoforms, UGT1A9 and UGT2B7 are predominantly involved in glucuronidation of NSAIDs [11,12]. All three NSAID-AGs tested formed a covalent adduct with UGT1A9 and 2B7. DIC-AG formed significantly lower amounts of covalent adducts with UGT2B7 than with UGT1A9. In contrast, ZOM-AG formed significantly higher amounts of covalent adducts with UGT2B7 than with UGT1A9. The capacity of diclofenac-AG to form covalent adducts with UGT1A9 or UGT2B7 was approximately 10 times higher than that of mefenamic acid-AG.

**Figure 5:**
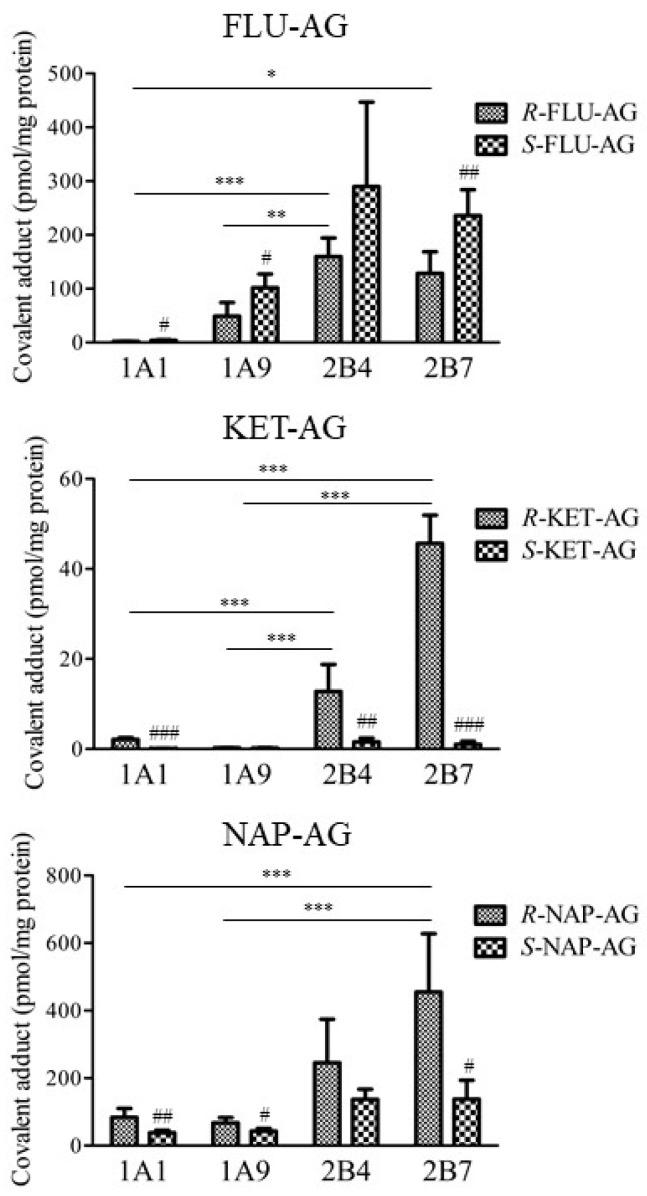
Covalent adduct formation of FLU-AG, KET-AG, and NAP-AG with UGT1A1, UGT1A9, UGT2B4, and UGT2B7. *R*- or *S*-NSAIDs-AG were incubated with respective UGT isomers for 6 h. The results are expressed as the mean ± SD of each group (*n* = 3–5). Statistical analysis was performed using the Bonferroni test. *, **, and *** indicate significant differences among UGT isoforms with *p* < 0.05, *p* < 0.01, and *p* < 0.001, respectively. #, ##, and ### indicate significant differences between *R* and *S* diastereomer with *p* < 0.05, *p* < 0.01, and *p* < 0.001, respectively.

AG of FLU, KET, and NAP as propionic acid derivatives were examined to quantitatively compare the formation of covalent adducts with UGT1A1, UGT1A9, UGT2B4, and UGT2B7 (Figure 5). Overall, the number of covalent adducts with UGT2B isoforms was higher than that with UGT1A isoforms. *R*- and *S*-FLU-AG, *R*-KET-AG, and *R*-NAP-AG formed relatively higher amounts of covalent adducts with UGT2B. Covalent adduct formation of *S*-FLU-AG with UGT1A1, UGT1A9, and UGT2B7 was significantly higher than that of *R*-FLU-AG. In contrast, *R*-KET-AG formed significantly more covalent adducts with UGT1A1, UGT2B4, and UGT2B7 than *S*-KET-AG. *R*-NAP-AG formed significantly more covalent adducts with UGT1A1, UGT1A9, and UGT2B7 than *S*-NAP-AG. In particular, KET-AG exhibited remarkably stereoselective formation of a covalent adduct with UGT2B. There was no formation of covalent adducts between the parent NSAIDs and UGTs (data not shown). There was no formation of covalent adducts of NSAIDs-AG in the incubation conditions without UGT proteins (data not shown).

**Figure 6:**
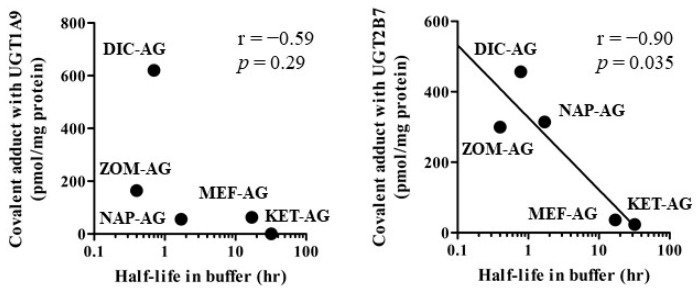
Correlation between the logarithm of half-lives in phosphate buffers (pH 7.4) and covalent adduct amounts of DIC-AG, MEF-AG, and ZOM-AG or average covalent adduct amounts of *R*- and *S*-KET-AG, or NAP-AG with UGT1A9 or UGT2B7. Pearson coefficient values (r) and *p*-values (*p*) were shown.

The correlation between the logarithm of half-lives in phosphate buffers (pH 7.4) reported in previous reports [13,14] and covalent adduct amounts of DIC-AG, MEF-AG, and ZOM-AG, or average covalent adduct amounts of *R*- and *S*-KET-AG, or NAP-AG with UGT1A9 or UGT2B7 were examined (Figure 6). A significant negative correlation (*p* = 0.035) between the half-lives of NSAIDs-AG and the amount of covalent adduct with UGT2B7, but not UGT1A9, was observed.

## 3. Discussion

In this study, we showed that NSAIDs-AG forms covalent adducts with organelle proteins and UGT isoforms. After the evaluation of covalent adduct formation with the proteins Cyt, Mic, Mit, and Nuc, the quantitative evaluation of covalent adducts of nine NSAIDs-AG with UGT isoforms was performed.

Although NSAIDs-AG is known to bind covalently with serum proteins such as HSA, covalent adduct formation with organelle proteins is not fully understood. The covalent adducts of DIC-AG with organelle proteins were approximately twice as high as those of *R*-KET-AG (Figure 3). Stereoselective covalent adduct formation of KET-AG with the proteins Cyt, Mit, and Nuc was observed; that is, higher amounts of covalent adducts of *R*-KET-AG were observed compared with those of *S*-KET-AG. Most 2-aryl propionic NSAIDs-AGs, including KET-AG, stereoselectively interact with HSA in reversible and irreversible manners [15,16]. Hayball et al. demonstrated that the hydrolysis half-life of *R*-KET-AG in plasma at physiological pH and temperature was shorter than that of *S*-KET-AG [15], suggesting a higher instability of *R*-KET-AG compared to *S*-KET-AG. Other reports demonstrated that acyl migration mainly contributed to the instability of NSAIDs-AG [14,17,18]. In actuality, Akira et al. showed a higher acyl migration rate of *R*-KET-AG compared with *S*-KET-AG [19]. The acyl migration is required to form irreversible binding [20]. Taken together, it is assumed that *R*-KET-AG forms higher amounts of covalent adducts with proteins of Cyt, Mit, and Nuc, probably due to the higher instability of *R*-KET-AG. Both DIC-AG and KET-AG tended to bind to the proteins of Cyt when compared to other organelle proteins. Inoue et al. demonstrated that DIC-AG may bind to 59 proteins of human hepatocytes, such as chaperone proteins; metabolic proteins related to glycolysis, alcoholysis, and lipidolysis; and mitochondrial proteins [21]. These results suggest that the covalent adducts of NSAIDs-AG form with a broad variety of organelle proteins. The covalent adducts with the Cyt and Nuc proteins were higher than those with the Mic and Mit proteins. Nucleophilic functional groups such as -NH_2_ and -OH of lysine, arginine, and serine in proteins covalently bind to NSAIDs-AG. Therefore, it can be presumed that Cyt and Nuc can abundantly include proteins that contain nucleophilic functional groups. However, some attention should be paid to the interpretation of the results of covalent binding of NSAIDs-AG to organelle-derived proteins, because the exact purity of extracted organelle was unclear.

Next, we examined covalent adduct formation with UGT, which is contained in proteins of Mic, given that the concentrations of NSAIDs-AG in the ER could be relatively high just after the formation of NSAIDs-AG by UGT. UGT1A1, UGT1A9, UGT2B4, and UGT2B7 are predominantly involved in the conversion of NSAIDs to NSAID-AG. DIC and ZOM are typical substrates for UGT2B7 [22,23,24]. MEF, FLU, and NAP are substrates for UGT1A9 and UGT2B7 [23,25], while KET is a substrate for UGT2B4 and UGT2B7 [23]. Regardless of the type of NSAIDs, the formation of covalent adducts of NSAIDS-AG with UGT isoforms was observed (Figure 4 and Figure 5), suggesting that NSAIDs-AG did not preferentially bind to UGT isoform-catalyzing glucuronidation of parent NSAIDs. DIC-AG, NAP-AG, and ZOM-AG, which have relatively short half-lives in buffers, formed higher amounts of covalent adducts with UGT2B7 (Figure 6). A previous report showed a good correlation between the amount of covalent adduct and HSA and a constant degradation rate [26]. AG of propionic acid derivatives such as FLU, KET, and NAP tend to bind to UGT2B isoforms compared with UGT1A isoforms (Figure 5). Compared to the composition of amino acids in UGT1A and UGT2B isoforms, the ratios of lysine, arginine, and serine in UGT2B isoforms were higher than when compared with those in UGT1A isoforms. In particular, there were approximately 1.5-fold differences (22.5% vs. 14.8%) in 20% amino acids from the N-terminus inside the ER. This difference possibly caused the formation of higher amounts of covalent adduct covalent amounts in UGT2B isoforms than in UGT1A9 isoforms. Understandably, the conformation of UGT in the ER determines the interaction between NSAIDs-AG and UGT. However, the detailed differences in covalent adduct formation between UGT1A and UGT2B cannot be fully explained because the crystal structure of UGT in the ER membrane is undetermined. In addition, UGT2B7 dimerizes with UGT1A1 and UGT1A9, localizes in the ER membrane [27], and interacts with CYP3A4 [28]. Thus, consideration should be given to the in vivo existence state of UGT in the ER membrane in further studies on covalent adduct formation of NSAIDs-AG with UGT.

There was a significant negative correlation between the logarithm of the half-lives of NSAIDs-AG in buffers and the covalent adduct amounts of DIC-AG, MEF-AG, and ZOM-AG, or average covalent adduct amounts of *R*- and *S*-KET-AG, or NAP-AG with UGT2B7. Correlation analysis was performed using the average of the covalent adduct amounts of diastereomers but not each diastereomer. Therefore, attention should be paid to the results of the correlation analysis. Acyl migration is required to form irreversible binding [20], and the rate of acyl migration is 10–20-fold faster than that of hydrolysis under in vitro physiological conditions [29]. Therefore, the rate of acyl migration is a primary determinant of the half-lives of NSAIDs-AG in phosphate buffers (pH 7.4). It is suggested that the more labile NSAID-AG forms higher irreversible binding to UGT.

There was a stereoselective formation of covalent adducts of NSAIDs-AG with endogenous proteins such as serum albumin and α1-acid glycoprotein [30]. Remarkable differences between diastereomers in covalent adduct formation were observed in KET-AG; that is, the amounts of covalent adducts of *R*-KET-AG with Cyt, Mit, Nuc, UGT1A1, UGT2B4, and UGT2B7 were higher than that of *S*-KET (Figure 3 and Figure 5). These results are related to the instability of the diastereomers. In many instances, acyl glucuronides of *R*-diastereomers of NSAIDs exhibit higher half-lives in buffers [13,15,31]. However, the conformational effects of stereoisomers on binding with UGT cannot be evaluated because the binding site of NSAIDs-AG to UGT in the ER membrane is undetermined. Therefore, a limitation of our study is that the determinant for the number of covalent adducts of NSAIDs-AG with UGT is not entirely clear, although these results provide comprehensive information on covalent adduct formation of NSAIDs-AG with endogenous proteins such as UGT.

Stereoselective interactions between NSAIDs-AG and endogenous proteins determine pharmacokinetics, although it is unclear whether the formation of covalent adducts with UGT has an impact on pharmacokinetics. For example, in the interaction between NSAIDs-AG and efflux transporter MRP2/4, *R*-isomers showed a higher inhibitory effect on the transport activity of MRP4 than MRP2, and *S-diastereomers* showed a higher inhibitory effect on the transporter activity of MRP2 than MRP4 [32]. The covalent adduct formation of AG with endogenous proteins affects protein function and occasionally leads to autoimmunity [6]. Therefore, further studies are needed to clarify the effects of covalent adduct formation of NSAIDs-AG with UGT on the metabolic activity of UGT.

## 4. Materials and Methods

### 4.1. Chemicals and Reagents

DIC, MEF, ZOM, FLU, KET, NAP, and protease inhibitor cocktail were purchased from Sigma-Aldrich Co., LLC. Software (St. Louis, MO, USA). The FLU enantiomers (*R*- and *S*-FLU), KET enantiomers (*R*- and *S*-KET), NAP enantiomers (*R*- and *S*-NAP), and DIC-d4 were obtained from Toronto Research Chemicals Inc. (Toronto, Canada). Corning Supersomes Human UGT1A1, UGT1A9, UGT2B4, and UGT2B7 were purchased from Corning, Inc. (Corning, NY, USA). The Pierce BCA Protein Assay Kit was purchased from Thermo Fisher Scientific Inc. (Waltham, MA, USA). RIPA lysis buffer, including protease inhibitors, was obtained from Santa Cruz Biotechnology (Dallas, TX, USA). All other chemicals and solvents were of MS grade or higher and commercially available.

### 4.2. Animals

Eight-week-old male Wistar rats were obtained from Japan SLC Inc. (Shizuoka, Japan). The rats were housed in an air-conditioned room at 24 ± 1 °C and at a relative air humidity level of 55 ± 10%, with a 12 h lighting schedule (7:00 a.m. to 7:00 p.m.) with free access to standard laboratory food (MF; Oriental Yeast Co., Ltd., Tokyo, Japan). The study protocol was approved by the Committee for the Care and Use of Laboratory Animals of the Faculty of Pharmacy of Kindai University (Osaka, Japan).

### 4.3. Subcellular Fractionation of Liver Homogenates

The livers were excised from the rats after they were euthanized with sodium pentobarbital after saline perfusion. The liver was homogenized with a homogenization buffer containing 50 mM Tris-HCl, pH 7.4, 150 mM potassium chloride, 2 mM ethylenediaminetetraacetic acid, and protease inhibitor cocktail, which was then centrifuged at 600× *g* for 10 min at 4 °C. The precipitate was collected as a crude nuclear fraction. Similarly, after the supernatant was centrifuged at 8000× *g* for 10 min at 4 °C, the corresponding precipitate was collected as a crude mitochondrial fraction. After the supernatant was ultracentrifuged at 105,000× *g* for 60 min at 4 °C, the supernatant and precipitate were collected as a cytosolic fraction (Cyt) and a microsomal fraction (Mic), respectively. The nuclear fraction (Nuc) was purified from the crude nuclear fraction via a sucrose density gradient centrifugation with 250 mM to 2.3 M sucrose in 1 M Tris-HCl, pH7.4, 1 M potassium chloride, and 1 M MgCl_2_ at 12,000× *g* for 30 min at 4 °C. The mitochondrial fraction (Mit) was purified from a crude mitochondrial fraction via a sucrose density gradient centrifugation with 1.1, 1.3, 1.56, and 1.6 M sucrose in 1 M Tris-HCl, pH7.4, 1 M potassium chloride, and 1 M MgCl_2_ at 29,000× *g* for 200 min at 4 °C. After RIPA lysis buffer was added to the subcellular fractions, the mixtures were sonicated for 30 s and were centrifuged at 14,000× *g* for 15 min at 4 °C. The protein concentrations in the supernatant of Cyt, Mic, Mit, and Nuc were measured using a BCA protein assay kit.

### 4.4. Preparation of NSAIDs-AG

All NSAIDs-AG used in this study were prepared biosynthetically in vitro from the respective parent drugs using rat liver microsomes as per the published methods [33,34]. The concentrations and purity of NSAIDs-AG were estimated via high-performance liquid chromatography (HPLC) after alkaline hydrolysis of NSAIDs-AG to parent NSAIDs. HPLC analysis was performed using a reverse-phase column (COSMOSIL 5C18-ARII, 4.6 mm × 150 mm, 5 μM, Nacalai Tesque Inc., Kyoto, Japan) and an Agilent 1200 series HPLC system equipped with a UV detector (Agilent Technologies, Inc., Santa Clara, CA, USA). The column temperature was set to 40 °C, and the autosampler was maintained at 10 °C. The mobile phase (50% methanol in 8.5 mM ammonium formate and 0.0075% formic acid) was pumped at a flow rate of 1 mL/min at a UV wavelength of 254 nm. The purities of the glucuronides (KET-AG, 100%; other NSAIDs-AG, >96%) were confirmed after cleavage of the respective parent drugs with β-glucuronidase and 1 M NaOH. The obtained NSAIDs-AGs were stored at −80 °C until use.

### 4.5. Covalent Adduct Formation of NSAIDs-AG with Organelle Proteins, UGT1A, and UGT2B Isoforms

To determine the effects of DIC-AG concentrations on covalent adduct formation, DIC-AG (120, 180, 300, or 600 μM) was incubated with the proteins of Mic (1 mg/mL) for 6 h at 37 °C. For the time course of covalent adduct formation of DIC-AG with the proteins of Mic, DIC-AG (180 μM) was incubated with the proteins of Mic (1 mg/mL) for 1, 3, 6, and 24 h at 37 °C. To determine the covalent adduct formation of DIC-AG, *R*-KET-AG, and *S*-KET-AG with the proteins Cyt, Mic, Mit, and Nuc, DIC-AG, *R*-KET-AG, and *S*-KET-AG (180 μM) were incubated with the proteins Cyt, Mic, Mit, and Nuc (1 mg/mL) for 6 h at 37 °C.

To determine the formation of covalent adducts of DIC-AG, MEF-AG, *R*-FLU-AG, *S*-FLU-AG, *R*-KET-AG, *S*-KET-AG, *R*-NAP-AG, and *S*-NAP-AG with UGT1A1, UGT1A9, UGT2B4, and UGT2B7, NSAID-AG (180 μM) were incubated with UGT (3.4 μM) for 6 h at 37 °C. The concentrations of UGT were set at 3.4 μM by the preliminary experiments on the linearity of covalent adduct formation.

The covalent adduct formation of NSAIDs-AG with organelle proteins and UGT was determined according to previous reports on NSAIDs-AG with albumin with some modifications [35]. After a deproteinization of the reaction mixture, methanol/diethyl ether (3:1) was added to the pellet. The pellet was washed 14 times and dried overnight at room temperature. The dried pellet was hydrolyzed in 200 μL of 0.2 M NaOH for 16 h to release the parent NSAIDs. The concentrations of the parent NSAIDs were measured using liquid chromatography–tandem mass spectrometry (LC-MS/MS).

### 4.6. Determination of NSAIDs Concentrations by LC-MS/MS Method

Aliquots of 10 μL were injected into the LC-MS/MS system. The LC-MS/MS equipment consisted of an LC system (UltiMate 3000 series, Thermo Fisher Scientific, Inc.) and TSQ Endura Triple Quadrupole Mass Spectrometer with electrospray ionization (Thermo Fisher Scientific, Inc.). Finnigan Xcalibur software (Thermo Fisher Scientific, Inc.) was used for data recording and analysis. Analysis was further performed using a reversed-phase column (COSMOSIL 5C18-MSII, 2.0 mm × 150 mm, 5 μm; Nacalai Tesque, Inc.). The column temperature was set to 40 °C, and the autosampler was maintained at 10 °C. The mobile phase (A; 30% methanol in 8.5 mM ammonium formate and 0.0075% formic acid, and B; 90% methanol in 8.5 mM ammonium formate and 0.0075% formic acid) was pumped at a flow rate of 0.2 mL/min. The initial mobile phase composition was maintained at 30% mobile phase B for 1 min, changed linearly to 90% mobile phase B from 1 to 8 min, maintained for 1 min, returned to 30% mobile phase B from 9 to 10 min, and maintained for 1 min for chromatographic column equilibrium. The MS scan was operated in positive ionization mode for DIC, MEF, ZOM, KET, and NAP, or negative ionization mode, for FLU. The conditions for electrospray ionization were set at 3500 V spray voltage for positive ionization mode and 2500 V spray voltage for negative ionization mode at 400 °C capillary temperature, and 300 °C vaporizer temperature with 40 arbitrary units of nitrogen sheath gas and 10 arbitrary units of auxiliary gas. The MS scan was operated in the positive or negative ionization mode. The selected reaction monitoring (SRM) mode was used, with argon as the collision gas at 1.5 mTorr. The mass resolution was set at 0.7 full-width at half-height (unit resolution). SRM transitions (precursor and product ions) were mass to charge ratios of (*m*/*z*) 296 > 214 for DIC (positive ionization mode), 294 > 250 for DIC (negative ionization mode), 242 > 224 for MEF, 292 > 139 for ZOM, 243 > 198 for *R*- and *S*-FLU, 255 > 209 for *R*- and *S*-KET, 231 > 185 for *R*- and *S*-NAP, and 302 > 220 for DIC-d4. The collision energies were 15 V for DIC (negative ionization mode), ZOM, MEF, FLU, KET, and NAP; 35 V for DIC (positive ionization mode); and DIC-d4. The concentrations of NSAIDs were determined from a single-point calibration in every assay for NSAIDs after confirmation of linearity between area and concentrations of NSAIDs.

### 4.7. Statistical Analysis

The significance of differences between mean values was determined by Student’s *t*-test or the Bonferroni test after analysis of variance and the correlation between the logarithm of half-lives in phosphate buffers (pH 7.4) and covalent adduct amounts of DIC-AG, MEF-AG, and ZOM-AG, or the average covalent adduct amounts of *R*- and *S*-KET-AG, or NAP-AG with UGT1A9 or UGT2B7 via Pearson’s correlation and regression analysis using GraphPad Prism software version 5 (GraphPad Software, Inc., La Jolla, CA, USA). Statistical significance was set at *p* < 0.05.

## 5. Conclusions

To our knowledge, this is the first comprehensive study of covalent adduct formation of NSAIDs-AG with UGT isoforms. The amounts of covalent adducts of ZOM-AG, FLU-AG, KET-AG, and NAP-AG with UGT2B isoforms were higher than those with the UGT1A isoforms.

## Data Availability

The datasets used and/or analyzed during the current study are available from the corresponding author on reasonable request.

## Abbreviations

AG	acyl-β-D-glucuronide
Cyt	cytosolic fraction
DIC	diclofenac
DILI	drug-induced liver injury
ER	endoplasmic reticulum
FLU	flurbiprofen
HPLC	high-performance liquid chromatography
HSA	human serum albumin
KET	ketoprofen
LC-MS/MS	liquid chromatographic-tandem mass spectrometry
MEF	mefenamic acid
Mic	microsomal fraction
Mit	mitochondrial fraction
*m*/*z*	mass to charge ratio
NAP	naproxen
NSAIDs	nonsteroidal anti-inflammatory drugs
Nuc	nuclear fraction
SRM	selected reaction monitoring
UGT	UDP-glucuronosyltransferase
ZOM	zomepirac

## References

[1] Kretz-Rommel A., Boelsterli U.A. (1994). Selective Protein Adducts to Membrane Proteins in Cultured Rat Hepatocytes Exposed to Diclofenac: Radiochemical and Immunochemical Analysis. Mol. Pharmacol..

[2] Nagao T., Tanino T., Iwaki M. (2003). Stereoselective Pharmacokinetics of Flurbiprofen and Formation of Covalent Adducts with Plasma Protein in Adjuvant-Induced Arthritic Rats. Chirality.

[3] Seitz S., Kretz-Rommel A., Oude Elferink R.P., Boelsterli U.A. (1998). Selective Protein Adduct Formation of Diclofenac Glucuronide Is Critically Dependent on the Rat Canalicular Conjugate Export Pump (Mrp2). Chem. Res. Toxicol..

[4] Kretz-Rommel A., Boelsterli U.A. (1994). Mechanism of Covalent Adduct Formation of Diclofenac to Rat Hepatic Microsomal Proteins. Retention of the Glucuronic Acid Moiety in the Adduct. Drug Metab. Dispos..

[5] Wang M., Gorrell M.D., McGaughan G.W., Dickinson R.G. (2001). Dipeptidyl Peptidase IV Is a Target for Covalent Adduct Formation with the Acyl Glucuronide Metabolite of the Anti-Inflammatory Drug Zomepirac. Life Sci..

[6] Tailor A., Waddington J.C., Meng X., Park B.K. (2016). Mass Spectrometric and Functional Aspects of Drug-Protein Conjugation. Chem. Res. Toxicol..

[7] Terrier N., Benoit E., Senay C., Lapicque F., Radominska-Pandya A., Magdalou J., Fournel-Gigleux S. (1999). Human and Rat Liver UDP-Glucuronosyltransferases Are Targets of Ketoprofen Acylglucuronide. Mol. Pharmacol..

[8] Bischer A., Zia-Amirhosseini P., Iwaki M., McDonagh A.F., Benet L.Z. (1995). Stereoselective Binding Properties of Naproxen Glucuronide Diastereomers to Proteins. J. Pharmacokinet. Biopharm..

[9] Presle N., Lapicque F., Fournel-Gigleux S., Magdalou J., Netter P. (1996). Stereoselective Irreversible Binding of Ketoprofen Glucuronides to Albumin. Characterization of the Site and the Mechanism. Drug Metab. Dispos..

[10] Shimada H., Kobayashi Y., Tanahashi S., Kawase A., Ogiso T., Iwaki M. (2018). Correlation between Glucuronidation and Covalent Adducts Formation with Proteins of Nonsteroidal Anti-Inflammatory Drugs. Eur. J. Pharm. Sci..

[11] Meech R., Hu D.G., McKinnon R.A., Mubarokah S.N., Haines A.Z., Nair P.C., Rowland A., Mackenzie P.I. (2019). The UDP-Glycosyltransferase (UGT) Superfamily: New Members, New Functions, and Novel Paradigms. Physiol. Rev..

[12] Jin C., Miners J.O., Lillywhite K.J., Mackenzie P.I. (1993). Complementary Deoxyribonucleic Acid Cloning and Expression of a Human Liver Uridine Diphosphate-Glucuronosyltransferase Glucuronidating Carboxylic Acid-Containing Drugs. J. Pharmacol. Exp. Ther..

[13] Sawamura R., Okudaira N., Watanabe K., Murai T., Kobayashi Y., Tachibana M., Ohnuki T., Masuda K., Honma H., Kurihara A. (2010). Predictability of Idiosyncratic Drug Toxicity Risk for Carboxylic Acid-Containing Drugs Based on the Chemical Stability of Acyl Glucuronide. Drug Metab. Dispos..

[14] Knadler M.P., Hall S.D. (1991). Stereoselective Hydrolysis of Flurbiprofen Conjugates. Drug Metab. Dispos..

[15] Hayball P.J., Nation R.L., Bochner F. (1992). Stereoselective Interactions of Ketoprofen Glucuronides with Human Plasma Protein and Serum Albumin. Biochem. Pharmacol..

[16] Dubois-Presle N., Lapicque F., Maurice M.H., Fournel-Gigleux S., Magdalou J., Abiteboul M., Siest G., Netter P. (1995). Stereoselective Esterase Activity of Human Serum Albumin toward Ketoprofen Glucuronide. Mol. Pharmacol..

[17] Hasegawa J., Smith P.C., Benet L.Z. (1982). Apparent Intramolecular Acyl Migration of Zomepirac Glucuronide. Drug Metab. Dispos..

[18] Hyneck M.L., Munafo A., Benet L.Z. (1988). Effect of PH on Acyl Migration and Hydrolysis of Tolmetin Glucuronide. Drug Metab. Dispos..

[19] Akira K., Taira T., Hasegawa H., Sakuma C., Shinohara Y. (1998). Studies on the Stereoselective Internal Acyl Migration of Ketoprofen Glucuronides Using 13C Labeling and Nuclear Magnetic Resonance Spectroscopy. Drug Metab. Dispos..

[20] Mizuma T., Benet L.Z., Lin E.T. (1999). Interaction of Human Serum Albumin with Furosemide Glucuronide: A Role of Albumin in Isomerization, Hydrolysis, Reversible Binding and Irreversible Binding of a 1-O-Acyl Glucuronide Metabolite. Biopharm. Drug Dispos..

[21] Inoue K., Mizuo H., Ishida T., Komori T., Kusano K. (2020). Bioactivation of Diclofenac in Human Hepatocytes and the Proposed Human Hepatic Proteins Modified by Reactive Metabolites. Xenobiotica.

[22] King C., Tang W., Ngui J., Tephly T., Braun M. (2001). Characterization of Rat and Human UDP-Glucuronosyltransferases Responsible for the in Vitro Glucuronidation of Diclofenac. Toxicol. Sci..

[23] Kuehl G.E., Lampe J.W., Potter J.D., Bigler J. (2005). Glucuronidation of Nonsteroidal Anti-Inflammatory Drugs: Identifying the Enzymes Responsible in Human Liver Microsomes. Drug Metab. Dispos..

[24] Gunduz M., Argikar U.A., Cirello A.L., Dumouchel J.L. (2018). New Perspectives on Acyl Glucuronide Risk Assessment in Drug Discovery: Investigation of In Vitro Stability, In Situ Reactivity, and Bioactivation. Drug Metab. Lett..

[25] Gaganis P., Miners J.O., Knights K.M. (2007). Glucuronidation of Fenamates: Kinetic Studies Using Human Kidney Cortical Microsomes and Recombinant UDP-Glucuronosyltransferase (UGT) 1A9 and 2B7. Biochem. Pharmacol..

[26] Bolze S., Bromet N., Gay-Feutry C., Massiere F., Boulieu R., Hulot T. (2002). Development of an in Vitro Screening Model for the Biosynthesis of Acyl Glucuronide Metabolites and the Assessment of Their Reactivity toward Human Serum Albumin. Drug Metab. Dispos..

[27] Yang Z.-Z., Li L., Wang L., Yuan L.-M., Xu M.-C., Gu J.-K., Jiang H., Yu L.-S., Zeng S. (2017). The Regioselective Glucuronidation of Morphine by Dimerized Human UGT2B7, 1A1, 1A9 and Their Allelic Variants. Acta Pharmacol. Sin..

[28] Takeda S., Ishii Y., Iwanaga M., Nurrochmad A., Ito Y., Mackenzie P.I., Nagata K., Yamazoe Y., Oguri K., Yamada H. (2009). Interaction of Cytochrome P450 3A4 and UDP-Glucuronosyltransferase 2B7: Evidence for Protein-Protein Association and Possible Involvement of CYP3A4 J-Helix in the Interaction. Mol. Pharmacol..

[29] Baba A., Yoshioka T. (2009). Structure-Activity Relationships for Degradation Reaction of 1-Beta-o-Acyl Glucuronides: Kinetic Description and Prediction of Intrinsic Electrophilic Reactivity under Physiological Conditions. Chem. Res. Toxicol..

[30] Shen Q., Wang L., Zhou H., Jiang H., Yu L., Zeng S. (2013). Stereoselective Binding of Chiral Drugs to Plasma Proteins. Acta Pharmacol. Sin..

[31] Volland C., Sun H., Dammeyer J., Benet L.Z. (1991). Stereoselective Degradation of the Fenoprofen Acyl Glucuronide Enantiomers and Irreversible Binding to Plasma Protein. Drug Metab. Dispos..

[32] Kawase A., Yamamoto T., Egashira S., Iwaki M. (2016). Stereoselective Inhibition of Methotrexate Excretion by Glucuronides of Nonsteroidal Anti-Inflammatory Drugs via Multidrug Resistance Proteins 2 and 4. J. Pharmacol. Exp. Ther..

[33] Iwaki M., Bischer A., Nguyen A.C., McDonagh A.F., Benet L.Z. (1995). Stereoselective Disposition of Naproxen Glucuronide in the Rat. Drug Metab. Dispos..

[34] Nozaki Y., Kusuhara H., Kondo T., Iwaki M., Shiroyanagi Y., Nakayama H., Horita S., Nakazawa H., Okano T., Sugiyama Y. (2007). Species Difference in the Inhibitory Effect of Nonsteroidal Anti-Inflammatory Drugs on the Uptake of Methotrexate by Human Kidney Slices. J. Pharmacol. Exp..

[35] Iwaki M., Ogiso T., Inagawa S., Kakehi K. (1999). In Vitro Regioselective Stability of Beta-1-O- and 2-O-Acyl Glucuronides of Naproxen and Their Covalent Binding to Human Serum Albumin. J. Pharm. Sci..

